# Generation of Scratches and Their Effects on Laser Damage Performance of Silica Glass

**DOI:** 10.1038/srep34818

**Published:** 2016-10-05

**Authors:** Yaguo Li, Hui Ye, Zhigang Yuan, Zhichao Liu, Yi Zheng, Zhe Zhang, Shijie Zhao, Jian Wang, Qiao Xu

**Affiliations:** 1 Fine Optical Engineering Research Centre, Chengdu 610041, China; 2 Research Center of Laser Fusion, China Academy of Engineering Physics, Mianyang 621900, China

## Abstract

Scratches are deleterious to precision optics because they can obscure and modulate incident laser light, which will increase the probability of damage to optical components. We here imitated the generation of brittle and ductile scratches during polishing process and endeavored to find out the possible influence of scratches on laser induced damage. Brittle scratches can be induced by spiking large sized abrasives and small abrasives may only generate ductile scratches. Both surface roughness and transmittivity are degraded due to the appearance of brittle scratches while ductile scratches make little difference to surface roughness and transmittance. However, ductile and brittle scratches greatly increase the density of damage about one order of magnitude relative to unscratched surface. In particular, ductile scratches also play an unignorable role in laser induced damage, which is different from previous knowledge. Furthermore, ZrO_2_ and Al_2_O_3_ polished surfaces appear to perform best in terms of damage density.

Fused silica glass has been utilized in many optical systems, particularly ultraviolet (UV) lasers, because of excellent transmittance over the IR-Vis-UV band. In high power lasers, optical components made of fused silica glass are usually used as transmitting lens and debris shield, etc^1^. These components stand very high laser energy fluence at 355/351 nm. The affordable laser fluence of bulk fused silica has been theoretically and experimentally shown to be ~100 J/cm^2^ at 355/351 nm at nanosecond regime^2^. However, almost all fused silica components are damaged permanently at far lower fluence, more often than not, <5 J/cm^2^ for polished surface^3^,^4^,^5^. The causes for such low damage threshold are ascribed to mechanical and chemical defects during the manufacturing of optical components, specifically scratches/cracks and contaminations^4^,^5^,^6^,^7^,^8^,^9^, among which scratches are the most influential factors that affect the laser damage performance of optical components in that they can accommodate absorbing substance and modulate incident laser^10^,^11^. The damage mechanism for nanosecond lasers are mainly thermal effect, that is, thermal heat due to the absorption of incident laser by absorbers will be deposited in local area and the absorbed heat will raise the temperature near the absorbers. Once the temperature exceeds the melting point or softening point of glass, the optical components will be damaged mechanically and irreversibly^12^,^13^,^14^,^15^,^16^. Therefore, scratches are the disastrous defects for laser optics and should be avoided as completely as possible^3^,^5^,^17^. But it is exceedingly difficult and prohibitive to obtain a large-aperture optics free from scratches and it is necessary to ascertain whether all kinds of scratches are detrimental to laser damage performance and how each kind of scratches affect laser damage characteristic of fused silica glass^18^,^19^. In this paper, we systematically studied the influence of ductile and brittle scratches generated artificially during polishing process on the damage performance of fused silica optical components. Our experimental results suggest that ductile scratches are dominant in quantity but they hardly affect the surface roughness and the transmittivity. The surface roughness remains ~1 nm (RMS) and transmittivity is still 93% at 355 nm even if there are numerous ductile scratches on the surface. In contrast, the surface roughness decreases to ~5 nm (RMS) and transmittivity drops to 89% for the surface with brittle scratches. However, both types of scratches have notable influence on the damage performance; they increase the density of damage over one order of magnitude at fluence of 8 J/cm^2^ (355 nm, 3 ns). The details are presented below in the following order: the next section involves the experimental procedure followed by the results and discussion of our investigation and last comes conclusion section.

## Methods

Samples used in the experiments were 50 mm in diameter and 5 mm thick and no obvious scratches were on the surface. The samples were polished with a polyurethane pad adhered onto a synthetic tin plate installed onto a lapping machine (FD-380XL, Fonda, China). The platen can rotate with respect to the central axis. The samples were located in a separator. Both the separator and the platen were driven independently. The polishing slurry was fed continuously at a flow rate of 10 mL/min. A dead load of 2.9 N was applied onto the backside of workpiece. The polishing time usually lasted 30 min and in some cases where scratches did not appear after 30 min lapping, the time was prolonged. Various combinations of slurries and polishing pads^20^ (Universal Photonics Inc., USA) were used in our experiments to generate scratches on glass surfaces in order to find out likely effect of polishing pad and polishing compound on laser damage performance. The details are tabulated in Table 1.

Abrasive sizes of polishing compounds were examined with a particle size analyzer (Mastersizer 3000, Malvern, UK). The morphology of the abrasives was inspected with a scanning electron microscope (Helios Nanolab 650, FEI, USA). The surface roughness was evaluated with an optical profiler (NewView 7200, Zygo, USA) and the transmittivity was tested with a spectrometer (Lambda 950, Perkin-Elmer, USA) over the range of 300 nm~1100 nm.

Damage density test was performed on a Nd:YAG laser damage testing system (Laser Zentrum Hannover e.V., Germany). The Gaussian laser pulse (8 ns@355 nm, beam waist 800 μm) was focused onto the rear surface of samples and the repetition rate was 10 Hz. The damage test protocol adopted was raster scan. The stage of sample holder moved at a certain speed so that each pulse overlapped with the pulses adjacent to them at FWHM (Full-Width-at-Half-Magnitude) to ensure that the scanned area was irradiated at nearly the same fluence. The sample surface was divided into 3~5 sub-regions which were illuminated at different laser fluence. Each sub-region was 10 mm × 10 mm in dimension. The detailed testing layout can be found elsewhere^21^,^22^.

The same area was monitored with an optical microscopy 500×(VHX-2000, Keyence, Japan) and stitched each image to form a large image prior to and following laser damage testing. If no cracks were found under high magnification, the scratches were viewed to be ductile, which are usually light color in the images. On the other hand, brittle cracks scatter light strongly, which will be dark in the image. In this way, the fractions of ductile and brittle scratches can also be quantified.

## Results and Discussion

### Abrasive size, Surface roughness, Scratches, and Transmittivity

**Figure 1:**
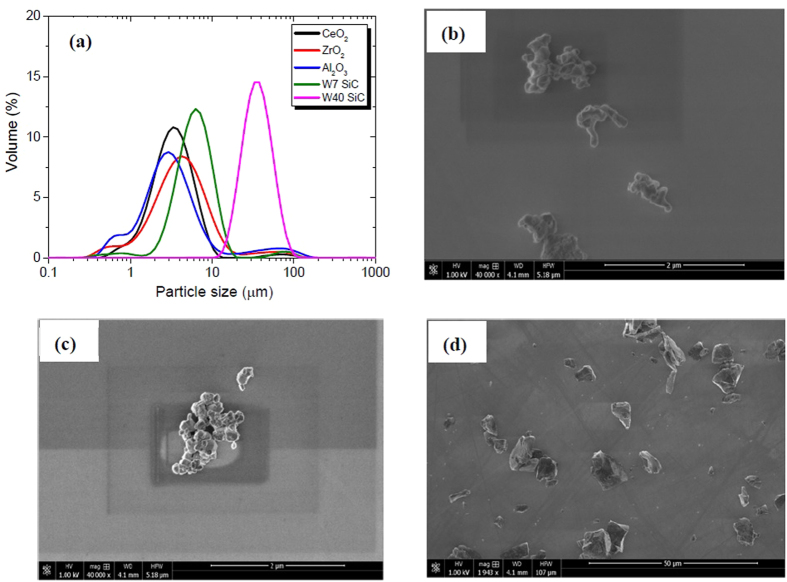
The size distribution and morphology of abrasives used in the experiments. (**a**) Size distribution of abrasives measured with a laser scattering size analyzer; (**b**) SEM image of Al_2_O_3_; (**c**) SEM image of CeO_2_; (**d**) SEM image of SiC W7. It is apparent that agglomeration forms in Al_2_O_3_ and CeO_2_ abrasives while agglomeration is seldom found in SiC.

The abrasives used in our experiments were observed with SEM and size analyzer and the particle diameter is found a bit different from the size provided by the manufacture. The size of SiC agrees well with the testing results while other abrasives do not. The nominal size of CeO_2_, ZrO_2_, and Al_2_O_3_ are all 0.3~0.5 μm, but the size analyzer suggests that the size all lies in 3~4 μm (D50). The reason may be the accumulation of micro-particles when the size is under 1 μm. Small particles are prone to agglomeration due to high relative surface area and high surface energy of small particles. Hence the abrasives were observed using SEM to verify our conjecture. We can understand that the SiC(W7, 7 μm) and SiC(W40, 40 μm) are dispersed very well whilst CeO_2_, Al_2_O_3_, and ZrO_2_ show apparent agglomeration (Fig. 1).

**Figure 2:**
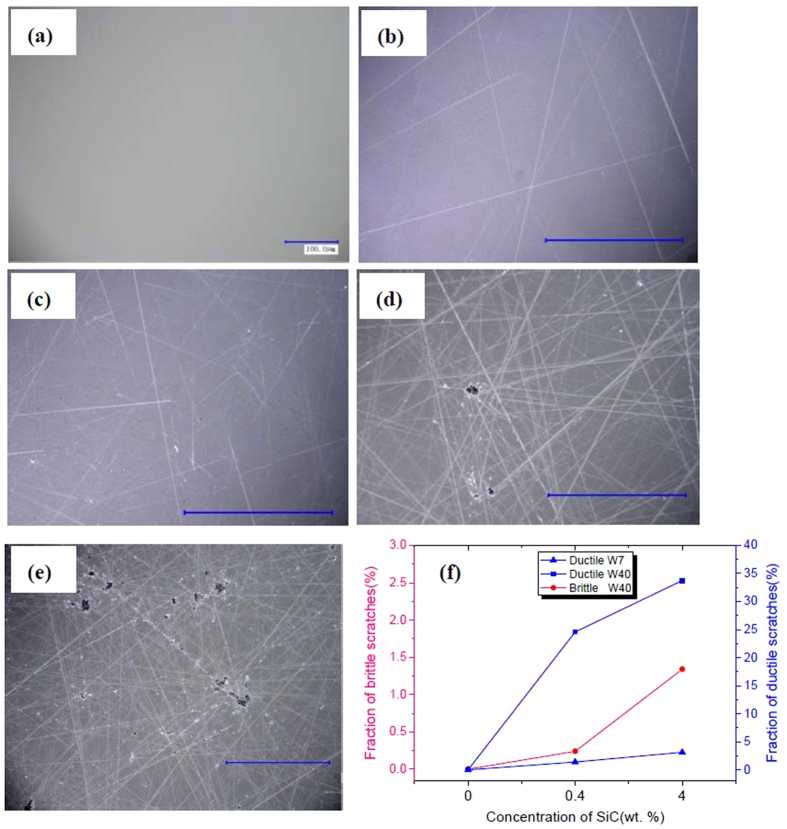
Surface morphology of sample A~E. (**a**) No scratches are found for sample A; (**b**) ductile scratches appear on the surface of sample B; (**c**) more ductile scratches occur on the surface of sample C; (**d**) ductile scratches and brittle scratches generate on sample D; (**e**) more brittle scratches and a vast number of ductile scratches happen on sample E; (**f**) increasing SiC concentration will give rise to more brittle and ductile scratches.

The surface roughness of each sample is listed out in Table 1 along with transimittivity and scratches. The surface was examined with optical microscopy (500× magnification) to find out whether scratches occur on the surface and whether the scratches are ductile or brittle. For the samples with obvious scratches, we quantified the scratches. The images were first binarized into white-black images and then the ratio of the scratch pixels to the whole pixels was considered to the quantity of scratches by using a software package ImageJ^23^. Comparing the results of scratches, we can see that CeO_2_ and Al_2_O_3_ did not induce scratches under normal polishing conditions, but ZrO_2_ sometimes may result in slight scratches which was also reported by other researchers^3^. It is known that complex chemical reactions between glass and CeO_2_ occur and a hydrated layer covers the surface of polished glass during the polishing process while only mechanical abrasion dominates the removal mechanism of glass when ZrO_2_ used as polishing compound^24^. It is the chemical reactions that accelerate the material removal rate during the polishing of glass, 1 μm/h for CeO_2_ abrasive versus 0.33 μm/h for ZrO_2_. After spiking SiC a kind of harder abrasive than CeO_2_, many scratches get visible (Fig. 2). The surface contains numerous ductile scratches on the surface polished with CeO_2_ doped with 7 μm SiC and the density of scratches gets high with increasing the concentration of SiC. However, almost no brittle scratches were found on the surface. On the other hand, there are a number of ductile scratches as well as some brittle ones on the surface processed by CeO_2_ plus 40 μm SiC. Likewise, the scratches including brittle and ductile become denser with increasing the concentration of SiC. Brittle scratches appear when 40 μm SiC abrasives were infiltrated into CeO_2_ slurry because increasing the size of SiC abrasives will decrease the number of abrasives bearing the downward load and therefore the load on a single abrasive will increase accordingly which will lead to brittle fractures when the load is in excess of a critical load to induce brittle fractures in fused silica.

**Figure 3:**
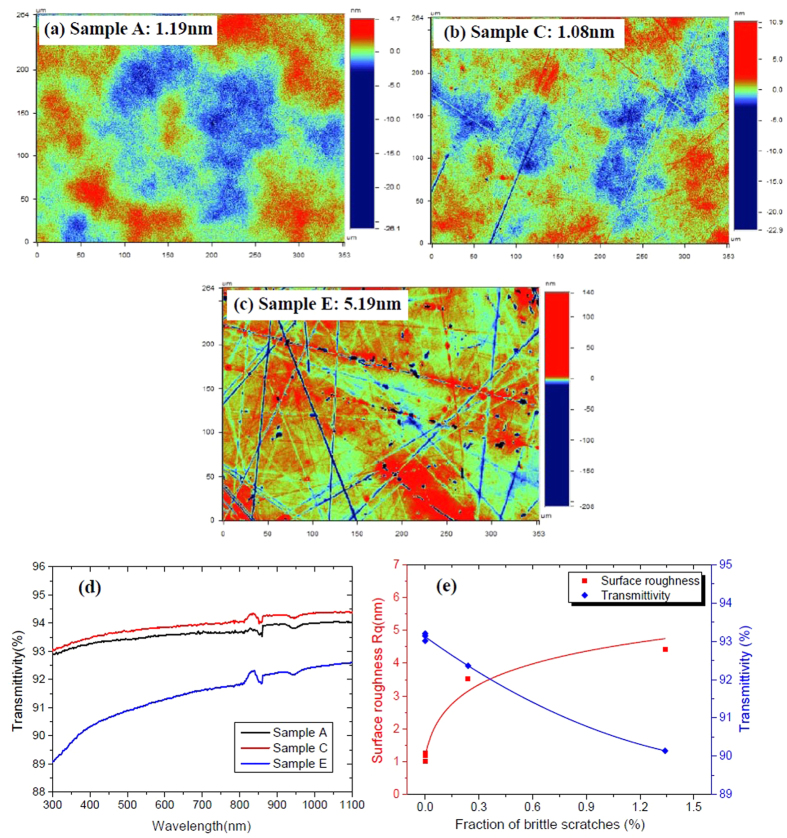
Surface micro-morphology of samples A, C & E. (**a**) surface roughness of sample A is 1.19 nm without scratches; (**b**) surface roughness of sample C is 1.08 nm with slight ductile scratches and the depth of scratches is ~20 nm; (**c**) roughness of sample E is over 5.19 nm with much deeper brittle and ductile scratches and the depth of the scratches is over 300 nm; (**d**) sample E has a lower transmittance than sample A&C over the UV-Vis-IR band; (**e**) the surface roughness and transmittance are strongly affected by brittle scratches.

Comparing the images of surface morphology and surface roughness in Fig. 3 and Fig. 2, it can be found that surface roughness is all ~1 nm except for the cases with 40 μm SiC since brittle scratches severely deteriorate surface quality and thereby surface roughness. The transmittivity spectra show that brittle scratches (Sample E) also strongly lower the transmittivity of fused silica in UV band (351/355 nm) from because they can scatter the incident light strongly and weaken the intensity of transmitted light (Fig. 3). Ductile scratches make much trivial difference to surface roughness and transmittivity as compared to brittle scratches and the transmittivity is only slightly reduced at 351/355 nm. Thus brittle scratches must be eradicated for precision optical components as only ~1.3% brittle scratches can increase surface roughness from ~1 nm to >4 nm and reduce the transmittance from >93% to <90%, which is undesirable in high power/energy laser systems.

### Damage performance

**Figure 4:**
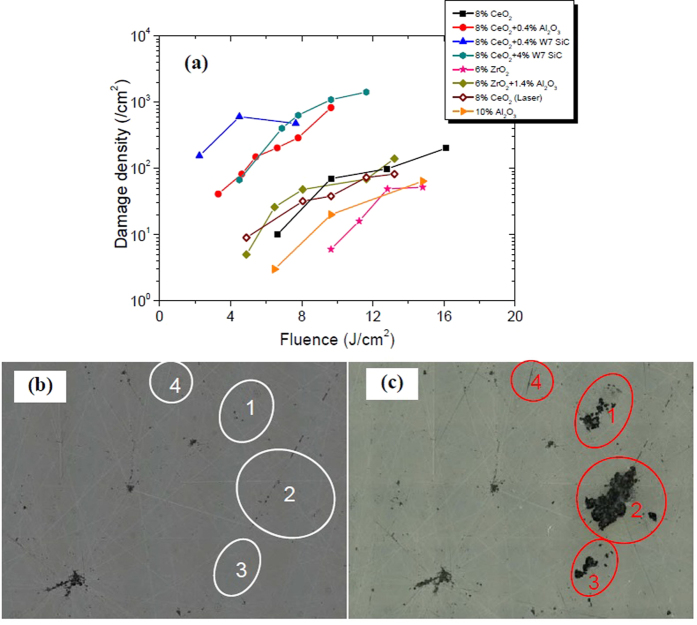
Damage performance of samples. (**a**) Damage density at varied laser fluence which shows ZrO_2_-polished sample is superior to other samples; (**b**) surface of sample D before raster scan damage testing; (**c**) sample D after damage testing, from which it is clean that both brittle and ductile scratches can cause laser-induced damage.

The samples were scanned with various energy fluences with 355 nm, 8 ns pulsed laser. The fluence was then converted to 3 ns with empirical rule^12^,^25^, and the fluence in the paper is all the converted one, i.e. 3 ns. The damage density was then extracted by comparing the defect density before and after testing. Each sample was scanned 3~6 regions so that damage density with fluence can be plotted. From the damage density results, it is clear that surfaces full of scratches are more sensitive to laser fluence. There are more mechanical defects at the intersecting points, e.g. micro-deformation of glass, micro-cracks, etc. and these defects will affect the laser damage performance of fused silica. Therefore, there is a higher probability that the laser induced ablation will be severer than other area. ZrO_2_ & Al_2_O_3_-polsihed samples perform better than other samples. The causes for the noticeable difference may be that Al_2_O_3_ and ZrO_2_ are not absorptive at 355 nm and with few scratches while CeO_2_ can greatly absorb the incident 355 nm laser, deposit the absorbed laser energy, heat the area locally and finally damage the fused silica sample. From the Fig. 4, field 1 & 2 were seriously damaged after laser illumination and there are brittle scratches in these two fields before damage testing. In spite of only ductile scratches in field 3 & 4, damage happened after raster-scan testing in the fields. Our results indicate that ductile scratches can also be damage precursors and can trigger damage to fused silica, which is different from previous results that ductile deformation may not be harmful to optical components in high power laser systems^18^,^19^.

## Conclusions

The artificial scratches were investigated to find out their possible effects on surface quality and laser damage. Various abrasives frequently used in optical manufacturing community were experimented. The results show that CeO_2_ is more efficient than Al_2_O_3_ and ZrO_2_ in polishing fused silica and CeO_2_, Al_2_O_3_ and ZrO_2_ are all capable of polishing out a smooth surface (surface roughness RMS ~ 1 nm). Adding SiC into CeO_2_ slurry will result in ductile and/or brittle scratches on polished surfaces, which depends on the size of abrasives added. Larger size will bring about ductile and brittle scratches while smaller abrasives may generate ductile scratches. Increasing the concentration of SiC will definitely raise the density of scratches. Furthermore, ductile scratches are found to have limited influence on surface roughness and transmittance while brittle scratches impact onto surface roughness and transmittance. From damage density results, it is found that ZrO_2_ and Al_2_O_3_ perform best in damage density and surfaces with numerous scratches usually damaged severely. Both ductile and brittle scratches can be precursors to laser damage and initiate catastrophic damage to optical components.

## Additional Information

**How to cite this article**: Li, Y. *et al*. Generation of Scratches and Their Effects on Laser Damage Performance of Silica Glass. *Sci. Rep.* **6**, 34818; doi: 10.1038/srep34818 (2016).

## Figures and Tables

**Table 1:** Details of polishing conditions for fused silica samples used in the experiments.

Sample	Slurry	Polishing pad^20^	Polishing time	Material removal rate (μm/h)	Surface roughness	Scratches		Transmittivity @355 nm
						Ductile	Brittle	
A	CeO_2_ wt.8% + Al2O_3_ wt.0.4%	LP-66	1.5 h	N/A	1.27 nm	0%	0%	93.139%
B	CeO_2_ wt.8% + SiC W7 wt.0.4%	LP-66	0.5 h	N/A	1.19 nm	1.39%	0%	93.006%
C	CeO_2_ wt.8% + SiC W7 wt.4%	LP-66	0.5 h	N/A	1.02 nm	3.135%	0%	93.204%
D	CeO_2_ wt.8% + SiC W40 wt.0.4%	LP-66	0.5 h	N/A	3.52 nm	24.55%	0.24%	92.363%
E	CeO_2_ wt.8% + SiC W40 wt.4%	LP-66	0.5 h	N/A	4.42 nm	33.72%	1.34%	90.1405%
F	ZrO_2_ wt.6%	LP-57	2.1 h	0.33	1.05 nm	1.476%	0%	92.9405%
G	ZrO_2_ wt.6% + Al_2_O_3_ wt.1.4%	LP-57	3.3 h	N/A	1.16 nm	1.493%	~0%	92.807%
H	Al_2_O_3_ wt.10%	LP-57	5.5 h	0.065	0.67 nm	0%	0%	92.978%
I	CeO_2_ wt.8%	LP-57	3 h	1.03	1.89 nm	0%	0%	92.954%
J	CeO_2_ wt.8%	LASER	3.5 h	0.96	1.43 nm	0%	0%	93.111%

Each sample was identical before polishing and polished under different conditions.

## References

[b1] Campbell J. H. . NIF Optical Materials and Fabrication Technologies: An Overview. Proc. SPIE 5341, 84–101 (2004).

[b2] Merkle L. D., Koumvakalis N. & Bass M. Laser-induced bulk damage in SiO_2_ at 1.064, 0.532, and 0.355 μm. J. Appl. Phys. 55, 772–775 (1984).

[b3] Menapace J. A. . Combined Advanced Finishing and UV-Laser Conditioning for Producing UV-Damage-Resistant Fused Silica Optics. Proc. SPIE 4679, 56–68 (2002).

[b4] Wang J. . Producing fused silica optics with high UV-damage resistance to nanosecond pulsed lasers. Proc. SPIE 9532, 95320H (2015).

[b5] Li Y., Yuan Z., Wang J. & Xu Q. Laser-Induced Damage Characteristics in Fused Silica Surface Due to Mechanical and Chemical Defects during Manufacturing Processes, to be published.

[b6] Li Y. . The Characteristics of Optics polished with a polyurethane pad. Opt. Express 16, 10285–10293 (2008). 18607437 10.1364/oe.16.010285

[b7] Li Y. . A method for evaluating subsurface damage in optical glass. Opt. Express 18, 17180–17186 (2010). 20721106 10.1364/OE.18.017180

[b8] Wang J., Li Y., Han J., Xu Q. & Guo Y. Evaluating subsurface damage in optical glasses. J. Eur. Opt. Soc. - Rapid Publ. 6, 11001 (2011).

[b9] Li Y. . Morphology and distribution of subsurface damage in optical fused silica parts: Bound-abrasive grinding. Appl. Surf. Sci. 257, 2066–2073 (2011).

[b10] Salleo A., Genin F. Y., Yoshiyama J., Stolz C. J. & Kozlowski M. R. Laser-induced damage of fused silica at 355 nm initiated at scratches. Proc. SPIE 3244, 341–347 (1998).

[b11] Suratwala T. I. . HF-Based Etching Processes for Improving Laser Damage Resistance of Fused Silica Optical Surfaces. J. Am. Ceram. Soc. 428, 416–428 (2011).

[b12] Bude J. . High fluence laser damage precursors and their mitigation in fused silica. Opt. Express 22, 5839–5851 (2014). 24663921 10.1364/OE.22.005839

[b13] Han J. . Phase explosion induced by high-repetition rate pulsed laser. Appl. Surf. Sci. 256, 6649–6654 (2010).

[b14] Huang Y. . Energy transmissivity of high-power nanosecond laser pulse focused on glass. Optik 121, 2213–2216 (2010).

[b15] Han J. . Effects of laser plasma on damage in optical glass induced by pulsed lasers. Opt. Eng. 51, 121809 (2012).

[b16] Duan T., Li Y. & Niu R. On the mechanism of multi-pulses induced damage in dielectrics. Optik 124, 1528–1531 (2013).

[b17] Shi F., Shu Y., Dai Y., Peng X. & Li S. Magnetorheological elastic super-smooth finishing for high-efficiency manufacturing of ultraviolet laser resistant optics. Opt. Eng. 52, 1–9 (2013).

[b18] Miller P. E. . Laser Damage Precursors in Fused Silica. Proc. SPIE 7504, 75040X (2009).

[b19] Miller P. E., Bude J. D., Suratwala T. I., Shen N. & Laurence T. A. Fracture-induced subbandgap absorption as a precursor to optical damage on fused silica surfaces. Opt. Lett. 35, 2702–2704 (2010). 20717429 10.1364/OL.35.002702

[b20] Polyurethane LP Unalon. http://universalphotonics.com/Default.aspx?tabid=102&prtype=1105&prid=490 (Date of access: 14/09/2016).

[b21] Ye H. . Post-processing of fused silica and its effects on damage resistance to nanosecond pulsed UV lasers. Appl. Opt. 55, 3017–3025 (2016). 27139869 10.1364/AO.55.003017

[b22] Ye H. . Laser induced damage characteristics of fused silica optics treated by wet chemical processes. Appl. Surf. Sci. 357, 498–505 (2015).

[b23] Rasband W. ImageJ software package. https://imagej.nih.gov/ij/index.html (Date of access: 14/09/2016).

[b24] Cook L. M. Chemical processes in glass polishing. J. Non-Cryst. Solids 120, 152–171 (1990).

[b25] Carr C. W., Trenholme J. B. & Spaeth M. L. Effect of temporal pulse shape on optical damage. Appl. Phys. Lett. 90, 041110 (2007).

